# Advanced Glycation End Products: Potential Mechanism and Therapeutic Target in Cardiovascular Complications under Diabetes

**DOI:** 10.1155/2019/9570616

**Published:** 2019-12-06

**Authors:** Ping Yang, Jian Feng, Qing Peng, Xing Liu, Zhongcai Fan

**Affiliations:** Department of Vasculocardiology, The Affiliated Hospital of Southwest Medical University, Luzhou, Sichuan 646000, China

## Abstract

The occurrence and development of cardiovascular complications are predominantly responsible for the increased morbidity and mortality observed in patients with diabetes. Oxidative stress under hyperglycemia is currently considered the initial link to diabetic cardiovascular complications and a key node for the prevention and treatment of diabetes-related fatal cardiovascular events. Numerous studies have indicated that the common upstream pathway in the context of oxidative stress in the cardiovascular system under diabetic conditions is the interaction of advanced glycation end products (AGEs) with their receptors (RAGEs). Therefore, a further understanding of the relationship between oxidative stress and AGEs is of great significance for the prevention and treatment of cardiovascular complications in patients with diabetes. In this review, we will briefly summarize the recent research advances in diabetes with an emphasis on oxidative stress and its association with AGEs in diabetic cardiovascular complications.

## 1. Introduction

Diabetes and its associated complications present a global burden in terms of human health and economics [1], of which the prevalence is rising at an exponential rate worldwide. According to data from the International Diabetes Federation (IDF), the currently estimated total number of 18- to 99-year-old diabetic patients is approximately 425 million globally. Moreover, the number of adult diabetes patients is expected to continue to increase over the next several decades due to aging, urbanization, and changes in diet and physical activity. The number of adult diabetes patients was expected to increase to 693 million by 2045 [2, 3]. Hyperglycemia and insulin resistance can affect various tissues and organs throughout the body, causing chronic complications of multiple systems and organs, especially the cardiovascular system [4, 5]. Pathological remodeling of the heart is characterized by left ventricular concentric hypertrophy and perivascular and interstitial fibrosis, leading to diastolic dysfunctions [6]. Diabetic macroangiopathy includes atherosclerosis of the aorta, coronary arteries, cerebral arteries, renal arteries, and peripheral arteries, while diabetic microangiopathy includes diabetic retinopathy and diabetic nephropathy [7, 8].

Both macro- and microvascular complications adversely affect the quality of life of patients with diabetes [9, 10]. The death risk from major adverse cardiovascular events in diabetic patients is higher than that in nondiabetic patients. Cardiovascular disease is more severe and extensive in the former than in the latter, with a worse prognosis and earlier onset. Patients with type 2 diabetes are 2-4 times more likely to develop heart failure than nondiabetic patients [11]. Approximately 70-80% of diabetes patients eventually die from cardiovascular complications [12]. Moreover, approximately 3/4 patients with type 2 diabetes have a variety of cardiovascular risk factors, such as hypertension, dyslipidemia, and obesity. The clusters of these risk factors can directly promote the occurrence of cardiovascular complications in diabetes [13].

Although diabetes treatment has undergone a transformation from simple control of hyperglycemia to multi-risk-factor management, the prevention and control of cardiovascular complications in this population still remain challenging. Sustained blood-glucose elevation is the initiating factor in the pathogenesis of diabetic cardiovascular complications. However, the mechanisms by which hyperglycemia can affect the cardiovascular system have not been adequately addressed.

Various hyperglycemia-elicited metabolic and hemodynamic derangements have been proposed to contribute to cardiovascular complications in diabetes [6]. The currently identified mechanisms include increased oxidative stress [14, 15], activation of protein kinase C (PKC) [16], chronic inflammation [17, 18], mitochondrial dysfunction [19], and activation of the renin-angiotensin system (RAS) [20]. Among these, increased oxidative stress is considered to be the initial core mechanism leading to diabetic cardiovascular diseases [21]. Therefore, understanding how oxidative stress is controlled in the context of cardiovascular complications is helpful for developing effective therapeutics against diabetes.

Advanced glycation end products (AGEs) are a general term for a class of heterogeneous compounds mainly derived from nonenzymatic saccharification (Maillard reactions) of reducing sugar on proteins, lipids, and nucleic acids. AGEs can increase the production of reactive oxygen species (ROS), thereby initiating intracellular oxidative stress [22]. Conversely, the increase in ROS production can in turn promote the production of AGEs, thereby forming a vicious circle between oxidative stress and AGEs. In this review, we will briefly summarize the recent research advances in *in vitro* and *in vivo* model systems of diabetes with an emphasis on oxidative stress and its association with AGEs in cardiovascular complications under diabetic conditions in an effort to provide some evidence for potential cardiometabolic-targeted therapies for diabetes.

## 2. Oxidative Stress

Oxidative stress is defined as an imbalance between oxidization and antioxidation, which subsequently leads to multiple negative effects on cellular metabolism. “ROS” is a general term for active substances composed of oxygen in the body or in the natural environment, including free radicals (hydroxyl, superoxide) and nonradicals (hydrogen peroxide, singlet oxygen molecules). They are continuously generated and eliminated during redox reactions in life activities. ROS generation mainly stems from nicotinamide adenine dinucleotide phosphate (NADPH) oxidase [23]. Other sources include xanthine oxidase (XO) and nitric oxide synthase (NOS) decoupling.

Normally, a proper amount of ROS as a signaling molecule is indispensable for regulation of transcription factors, expression of apoptosis genes, and antibacterial and anti-inflammatory effects. During pathological conditions, however, when the ROS level exceeds the buffering capacity of antioxidant enzymes and antioxidants, the balance between oxidation and antioxidation shifts the trend to oxidization, resulting in oxidative stress [24]. It has been reported that increased ROS levels can stimulate mitogen-activated protein kinase (MAPK), tyrosine kinase, Rho kinase, and transcription factor (NF-*κ*B, AP-1, and HIF-1) activation [25–27]. Furthermore, ROS can inactivate protein tyrosine phosphatase (PTP), increase the intracellular free calcium ion concentration, and modulate the expression and activation of proinflammatory genes [28]. Changes in these intracellular signals can lead to endothelial dysfunction and myocardial remodeling.

## 3. Oxidative Stress in Diabetic Cardiovascular Complications

Increased oxidative stress is considered the initial core mechanism leading to diabetic cardiovascular diseases [21, 22]. In diabetic cardiovascular complications, NADPH oxidase is activated under conditions of hyperglycemia [29], catalyzing the formation of superoxide anions, and the superoxide anion undergoes a series of reactions to convert to hydroxyl groups, resulting in excessive ROS synthesis and subsequently leading to oxidative stress. In addition, the antioxidant capacity of the defense system, including enzymatic antioxidants (superoxide dismutase), nonenzymatic antioxidants (vitamin C, coenzyme Q10), and metal complexes (copper-binding proteins), is decreased by hyperglycemia [30, 31]. Oxidative stress triggers inflammation, endothelial dysfunction, cardiomyocyte hypertrophy and apoptosis, and myocardial fibrosis, which subsequently lead to decreasing left ventricular compliance, diastolic dysfunction, and finally heart failure, arrhythmia, and/or even sudden cardiac death.

### 3.1. Oxidative Stress and Inflammation

As previously reported, oxidative stress and inflammation interact with each other to promote diabetic cardiovascular complications [32]. ROS directly or indirectly activate NF-*κ*B, transforming growth factor-*β* (TGF-*β*), MAPK, protein kinase C (PKC), stress-activated protein kinase, etc. They thereby trigger inflammation and myocardial fibrosis in the cardiovascular system in diabetes [33, 34]. The expression of inflammatory factors such as tumor necrosis factor-*α* (TNF-*α*) and interleukin-6 (IL-6) was demonstrated to promote cardiac fibroblast proliferation, thereby increasing collagen synthesis and ultimately leading to myocardial fibrosis [35]. Inflammatory factors are in turn considered to increase ROS [36]. NF-*κ*B can increase the expression of inducible nitric oxide synthase (iNOS), which promotes the generation of nitric oxide. Excessive nitric oxide and peroxyl radicals react to form peroxynitrate, which subsequently increases mitochondrial permeability and ROS production.

### 3.2. Oxidative Stress and Endothelial Cell Dysfunction

In addition, the sustained elevation of blood glucose can cause endothelial cell dysfunction [37, 38]. Accumulated superoxide and nitric oxide interact rapidly to form a highly active intermediate, peroxynitrite. Peroxynitrite is a strong cytotoxic oxidant that can cause nitrosylation, nitration, and oxidative damage of biomolecules such as proteins, lipids, and DNA in endothelial cells [39, 40]. Simultaneously, peroxynitrite can cause the unfolding of endothelial NOS to form superoxide, and superoxide can continue to react with nitric oxide to form peroxynitrite, forming a vicious circle [41].

### 3.3. Oxidative Stress and Cardiac Hypertrophy

Oxidative stress also plays a key role in promoting cardiac hypertrophy in diabetes [42, 43]. Under the condition of hyperglycemia, ROS activate neurohumoral mechanisms such as the renin-angiotensin-aldosterone system, endothelin-1, and the sympathetic nervous system [44, 45]. Excessive activation of the renin-angiotensin-aldosterone system can induce cardiac hypertrophy and abnormal cardiac functions [46]. Studies have shown that the angiotensin-converting enzyme inhibitor lisinopril can inhibit 8-hydroxydeoxyguanosine and hydroxyl radicals in cardiomyocytes in diabetic rats and thus alleviate cardiac inflammation, fibrosis, and hypertrophy [47].

### 3.4. Oxidative Stress and Mitochondrial Dysfunction

Moreover, ROS-mediated activation of mitochondrial uncoupling proteins, increased proton leakage, and oxidative phosphorylation uncoupling can result in reduced adenosine triphosphate (ATP) production, thereby inducing cardiomyocyte apoptosis [48, 49]. The oxidative phosphorylation respiratory chain enzyme complex is composed of mitochondrial DNA and nuclear DNA-encoding subunits involved in the oxidative phosphorylation of mitochondria [50, 51]. On the one hand, increased ROS can directly damage mitochondrial DNA and membranes [52]. In addition to changes in mitochondrial ATP production, abnormalities in mitochondrial-surface membrane ion channels and sarcomere-associated proteins can also cause cardiac dysfunction [53]. The calcium signaling pathway is a prerequisite for myocardial cell contraction and relaxation. The sarcoplasmic calcium pump is an important component of the calcium signaling pathway, which relaxes cardiomyocytes by isolating calcium ions. ROS can cause dysfunction of the sarcoplasmic reticulum, leading to the accumulation of calcium ions in the cells, followed by cardiac dysfunction, arrhythmia, and heart failure [54].

**Figure 1:**
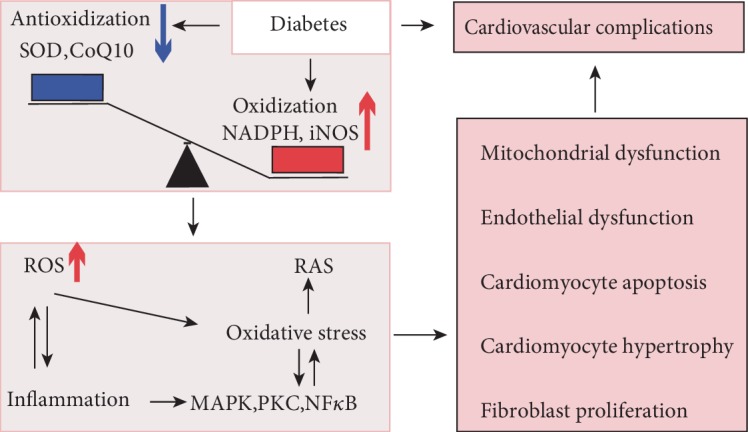
Oxidative stress plays an important role in diabetic cardiovascular complications. An impaired balance between oxidization and antioxidant activity in the diabetic cardiovascular system results in more pronounced ROS generation and oxidative stress. Oxidative stress interacts with inflammation and neurohumoral mechanisms, thereby promoting mitochondrial dysfunction, endothelial dysfunction, cardiac fibrosis, and hypertrophy.

All the above studies have shown that oxidative stress in the heart can cause endothelial and myocardial metabolic abnormalities through inflammation, mitochondrial damage, glucose metabolism disorders, and other mechanisms, eventually leading to myocardial contractile and diastolic dysfunction or even heart failure. Moreover, the abnormal elevation of oxidative stress can further cause glucose metabolism disorders, thereby creating a vicious circle if not controlled (Figure 1).

## 4. AGE Metabolism in Diabetic Cardiovascular Complications

The nonenzymatic glycosylation reaction, also known as the Maillard reaction, was proposed by Maillard in the early 20^th^ century [55]. AGEs are highly heterogeneous and exist in many different forms *in vivo*. Typical AGEs include pentosidine, carboxymethyl lysine (CML), carboxyethyl lysine (CEL), pyralline (Pyr), argpyrimidine (ArgP), and cross-linked AGEs [56, 57]. Once synthesis is finished, AGEs can accumulate with aging and can be used as a biomarker of aging [58]. AGEs function mainly through binding to specific receptors [59]. Currently identified receptors for AGEs include RAGE, macrophage scavenger receptors type I and type II, oligosaccharyl transferase-48, 80K-H phosphoprotein, and galectin-3, among which, RAGE is the major one [60]. The downstream targets of RAGE include NADPH oxidase, MAPK, extracellular signal-regulated kinase 1/2 (ERK1/2) and p38-mediated signaling pathways, and ultimately NF-*κ*B [61, 62].

Generally, the Maillard reaction occurs in some slower-renewing proteins, such as type IV collagen, laminin, and elastin, and the rate of AGE generation is relatively slow [63]. However, the formation and accumulation of AGEs have been known to progress at an accelerated rate in diabetes [64]. On the one hand, sustained hyperglycemia causes more rapid nonenzymatic glycosylation of the abovementioned proteins and results in the increase in AGEs. On the other hand, AGEs are scarcely degraded and remain for a long time in tissues even if glycemic control is improved [65]. The clearance of AGEs is mediated by specific receptors on macrophages via internalization or by extracellular proteolytic systems that decompose them into relatively lower molecular weight AGEs, which are finally cleared by the kidneys [66]. When nephritic insufficiency occurs in diabetes, AGEs increase in the circulation and further aggravate renal dysfunction [67]. Therefore, previous studies have paid more attention to the relationship between AGEs and diabetic nephropathy. Studies have confirmed that plasma AGE levels are closely related to the development of diabetic glomerular sclerosis, tubulointerstitial fibrosis, and mesangial cell proliferation [68, 69].

Increasing evidence indicates that AGEs are also involved in the occurrence and development of cardiovascular diseases. The increase in plasma AGEs is more pronounced in diabetic patients with coronary heart disease than in patients without coronary heart disease. Nin et al. [70] confirmed that plasma AGE levels are associated with all-cause mortality in fatal or nonfatal coronary artery disease. Steine et al. and Berg et al. [71, 72] found that left ventricular dysfunction in patients with type 1 diabetes is associated with plasma AGE levels. Jia et al. [73] also found that the level of tissue AGEs was independently associated with cardiac systolic dysfunction in diabetic patients with heart failure compared with diabetic patients without heart failure. Spadaccio and colleagues [74] showed that the risk of restenosis in diabetic patients with high plasma AGEs who undergo percutaneous coronary intervention (PCI) is relatively higher.

## 5. The Mechanism of AGEs in Diabetic Cardiovascular Complications

**Figure 2:**
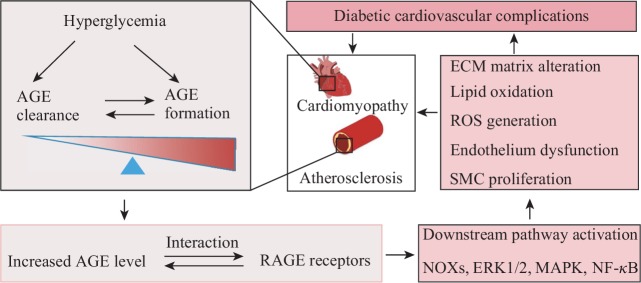
AGEs play an important role in diabetic cardiovascular complications. Under the condition of hyperglycemia, the balance between AGE formation and clearance is impaired in the diabetic cardiovascular system which thereby result in increased AGEs. AGEs interact with AGE receptors and then promote diabetic cardiovascular complications.

The effects of AGEs in the diabetic cardiovascular system are mediated by receptor-dependent and nonreceptor-dependent pathways (Figure 2). First, endothelial cell dysfunction is the starting event of atherosclerosis. AGEs can directly modify extracellular matrix proteins of endothelial cells, including type IV collagen and laminin [75, 76]. This process destroys the normal structure and function of blood vessels, and cardiac fibrosis is accelerated [77]. AGEs not only damage endothelial cells but also induce apoptosis and dysfunction of endothelial progenitor cells [78]. Ueda et al. [79] reported that serum AGE levels are independent risk factors for the number and function of circulating endothelial progenitor cells. In addition, AGEs can directly stimulate the production of vascular endothelial cell growth factor (VEGF), leading to increased vascular permeability or even vascular wall edema [80].

Additionally, circulating AGEs can increase lipid oxidation and deposition in atherosclerotic plaques and promote the infiltration of macrophages and T cell migration and proliferation, thereby promoting atherosclerosis [81]. AGE-induced LDL glycosylation results in the blocking of receptor-mediated LDL removal. Furthermore, increased glycosylation reduces the cholesterol reverse transport ability of HDL, thus promoting the deposition of lipids in blood vessel walls and resultant plaque formation [82, 83]. Recent studies have also shown that the binding of AGEs to the platelet membrane receptor CD36 induces thrombus formation, which may be an important mechanism by which AGEs promote cardiac ischemic events in diabetic patients [84].

In addition, a recent study confirmed that the AGE-RAGE axis interacts with the RAS, which contributes to the proliferation of cardiac fibroblasts and cardiomyocyte hypertrophy in diabetes as well [85]. Moreover, it was reported that AGEs can upregulate RAGE expression via the activation of NF-*κ*B [86]. As mentioned before, activated NF-*κ*B binds to specific DNA sequences, regulating corresponding gene transcription and accelerating the emergence of cardiovascular complications. It is conceivable that the positive feedback loops between AGEs and RAGE-downstream pathways could create a vicious cycle, thus promoting cardiovascular complications in diabetes.

## 6. Crosstalk between AGEs and Oxidative Stress in Diabetic Cardiovascular Complications

Oxidative stress and the AGE-RAGE axle pathway are not independent processes. Accumulating evidence has suggested that the crosstalk between AGE-RAGE and oxidative stress plays an important role in the context of cardiovascular complications of diabetes [64]. AGE-RAGE interaction results in the activation of diverse signal transduction cascades and downstream pathways such as MAPK, ERK1/2, p38, and NF-*κ*B, thereby including the generation of ROS and accelerating oxidative stress and the emergence of cardiovascular complications in diabetes [87, 88]. Blockade of RAGEs attenuates vascular oxidative stress and the development of atherosclerosis.

Tang et al. reported that AGEs can activate NF-*κ*B to increase the expression of inducible NO synthase (iNOS) through RAGE/RhoA/ROCK-mediated and AMPK-mediated signaling pathways, which subsequently promote the generation of NO in endothelial cells [89]. Hegab et al. found that treatment of cardiomyocytes with AGEs for 24 h significantly increased ROS production [90]. Chen et al. suggested that AGEs can induce oxidative stress through the Sirt1/Nrf2 axis by interacting with RAGEs under diabetic conditions [91]. AGEs were also reported to increase the expression and activity of NADPH oxidase in endothelial cells, which is an important source of oxidative stress in diabetic cardiovascular complications [92, 93]. Increased NADPH oxidase activity results in the generation of ROS and the depletion of cellular antioxidants such as glutathione, glutathione peroxidase, superoxide dismutase, and catalase.

**Figure 3:**
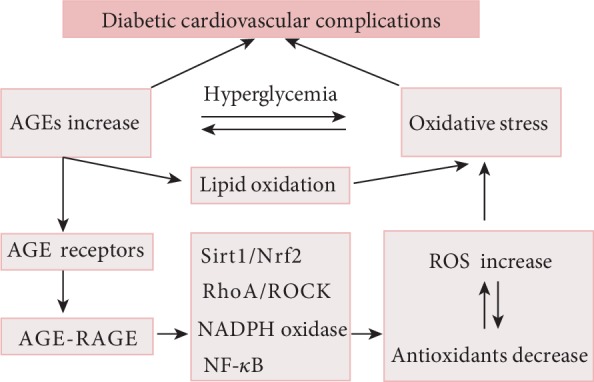
The interaction between AGE-RAGE axle and oxidative stress plays an important role in diabetic cardiovascular complications. On the one hand, AGE-RAGE axle activation can result in the activation of diverse signal transduction cascades, thereby including the generation of ROS and accelerating oxidative stress. On the other hand, excessive oxidative stress can in turn accelerate the generation of AGEs in the diabetic cardiovascular system.

It was reported that AGEs are involved in a vicious cycle of oxidative stress [94]. AGEs modulate oxidative stress, and excessive oxidative stress can in turn accelerate the generation of AGEs [95] such as CML [96]. Taken together, these data suggest that the crosstalk between the AGE-RAGE axis and oxidative stress is highly involved in the context of diabetic cardiovascular complications (Figure 3). A deeper study of this relationship will facilitate the designing of new drugs and provide new prospects and methods for the prevention and treatment of diabetes and its cardiovascular complications. Drugs that deplete AGEs in the cardiovascular system or block their interaction with oxidative stress may be proper candidates.

## 7. Current and Future Therapies against Diabetic Cardiovascular Complications

Lifestyle changes, balanced energy intake, and glucose- and lipid-lowering drugs are the currently available treatment strategies for diabetes patients with cardiovascular complications [97]. A detailed description of therapeutic approaches for diabetes with cardiovascular complications is beyond the scope of this review. Since AGEs, oxidative stress, and their interactions are highly related to the progression of diabetic cardiovascular complications, therapies that involve AGEs and oxidative stress may help reduce the cardiovascular complications in diabetic patients (Table 1).

### 7.1. Traditional Antidiabetic Agents

Oscillating glucose is more deleterious to endothelial function and oxidative stress in type 2 diabetic patients [98, 99]. Therefore, the key point for preventing and delaying the occurrence and development of diabetic cardiovascular complications lies in blood-glucose control [100]. In theory, a drug that lowers blood sugar confers cardiovascular protection, but actually, selective antidiabetic agents are limited. In contrast, some antidiabetic drugs even increase the risk of death from cardiovascular disease [101].

The UKPDS posttrial study and DCCT/EDIC showed that intensive blood-glucose control not only reduces the risk of microvascular disease in diabetes patients but also significantly reduces the total mortality rate of cardiovascular disease [102]. Intensive blood-glucose control with metformin is currently accepted to reduce the risk of cardiovascular disease in diabetes [103]. Its cardiovascular protection effect was attributed to the antioxidant properties that lead to the reduction of XO activity and lipid peroxidation in patients with type 2 diabetes [104]. Treatment of diabetic rats with metformin was also found to decrease the plasma levels of AGEs [105], thereby reducing oxidative stress and cardiac remodeling.

However, the conclusion of three other intensive hypoglycemic trials, ADVANCE, ACCORD and VADT, suggested that it may be difficult to effectively reduce cardiovascular risk in patients with type 2 diabetes simply by intensive glucose control [106]. Intensive hypoglycemic therapy increases the risk of hypoglycemia, which has been demonstrated to be significantly correlated with severe cardiovascular events in diabetes patients [107]. Moreover, since rosiglitazone was reported to be associated with a significant increase in the risk of myocardial infarction and death from cardiovascular events [108], the concern regarding the cardiovascular safety of antidiabetics has markedly increased even though, subsequently, the RECORD trial emphasized that rosiglitazone does not increase the risk of cardiovascular events [109].

### 7.2. Antioxidants

According to the “metabolic memory” theory, hyperglycemia-induced metabolic changes will last for a long time even after the blood-glucose level returns to normal [106, 110]. Clearly, oxidative stress is considered the key cause of cardiovascular complications of diabetes. Therefore, antioxidant therapy and hypoglycemic therapy are equally important.

Antioxidants such as vitamins and vitamin analogs are widely used clinically. Vitamin C is a water-soluble vitamin that has significant antioxidant effects. Vitamin C infusion was reported to improve endothelial function and cardiac diastolic function. However, it did not alter the exercise capacity in type 2 diabetes [111]. Vitamin E can reduce lipid peroxidation in patients with noninsulin-dependent diabetes, which is important for the treatment of early diabetic cardiovascular complications [112]. Specifically, in patients with haptoglobin genotype-2 (Hp2-2), vitamin E has been shown to be associated with an approximately 35% reduction in cardiovascular diseases in both type 1 diabetes and type 2 diabetes [113, 114]. This reduction was mediated partly by an improvement in the function of HDL. However, some clinical trial data suggest that vitamin E does not affect the development of cardiovascular diseases in some patients [115]. For vitamin E to be clinically used in diabetes, an additional large prospective study will be needed.

NADPH oxidase is the major source of ROS in oxidative stress under the condition of diabetes. Since angiotensin II can activate NADPH oxidase to increase oxidative stress through the AT1 receptor [116, 117], the administration of angiotensin-converting enzyme inhibitors (ACEIs) and angiotensin receptor blockers (ARBs) is currently the regular treatment for diabetes with cardiovascular complications. Statins were first known to reduce cholesterol synthesis through competitive inhibition of hydroxymethyl glutarate mononyl coenzyme A (HGM-CoA) reductase. An increasing number of studies have revealed that statins work as antioxidants beyond their lipid-lowering effect [118, 119]. In patients receiving different treatments, the antioxidant state is independently affected but is significantly more pronounced in patients on statins [120]. This effect is mediated mainly by reducing the expression of NADPH oxidase subunit and increasing the expression of antioxidant enzyme [121].

### 7.3. AGE-RAGE Axis Inhibitors

Pharmacological inhibitors of AGEs include aminoguanidine (AG), pyridoxamine (a natural vitamin B6 derivative), benfotiamine, ACEIs, ARBs, statins, N-phenylthiazole bromide (ALT-711), and thiazolidinediones [122–124]. AG is a nucleophilic hydrazine complex that inhibits AGE formation by combining early glycosylation, glycogen oxidation products, acetaldehyde products, etc., subsequently attenuating AGE and ROS formation both *in vivo* and *in vitro* [125]. However, AG has been limited in further clinical usage because it interferes with several important regulatory systems and has yielded toxic side effects (such as flu-like symptoms, anemia, and gastrointestinal reactions) in clinical trials.

Reactive carbonyl compounds are precursors for the formation of AGEs, which significantly accelerate the formation of AGEs. Pyridoxamine acts as a nucleophilic compound that scavenges carbonyl compounds and is a possible mechanism by which pyridoxamine inhibits the formation of AGEs [126]. Nagai et al. [127] suggested that some metal ions, such as copper ions, can participate in the autooxidation of glucose and early glycation products, playing an important role in the formation of AGEs. Triethylenetetramine (TETA) was reported to induce antidiabetic changes by targeting these copper-mediated pathogenic mechanisms [31].

N-Phenylthiazole bromide (such as ALT-711 and TRC4186) can cleave the protein cross-linking structure in AGEs and reduce the accumulation of tissue AGEs. *In vitro*, collagen that cross-linked with AGEs incubated with ALT-711 was found to be more rapidly broken down by metalloproteinases [128]. Resveratrol treatment significantly reduces oxidative stress in the kidneys of rats with diabetes by downregulating RAGE [129]. Irisin alleviates AGE-induced inflammation and endothelial dysfunction by inhibiting ROS-NLRP3 inflammasome signaling [130]. However, the clinical application of the abovementioned agents in diabetes needs further study.

The molecular structure of RAGE includes its extracellular domain, transmembrane structure, and intracellular structure. Soluble RAGE (sRAGE) only contains the extracellular segment and can competitively combine with AGEs but cannot complete signal transduction, thus blocking the harmful effects of AGEs [131]. Studies have demonstrated that a high plasma level of sRAGE is independently associated with a low recurrence of atrial fibrillation after catheter ablation in diabetic patients [132]. AGEs and RAGE are increased in the atherosclerosis plaques in apoE^−/−^ mice, whereas sRAGE treatment significantly reduces these changes [133]. Another study found that sRAGE can stabilize plaque and inhibit inflammatory factors such as cyclooxygenase-2 (COX-2), VCAM-1, and monocyte chemoattractant protein-1 (MCP-1), thereby reducing endothelial cell dysfunction [134].

### 7.4. New Hypoglycemic Agents

Although a series of drug candidates was reported to reduce oxidative stress and/or the AGE-RAGE axis, therapeutics that adequately address diabetic cardiovascular injuries have yet to be established clinically. Many trials of hypoglycemic therapy have also failed to prove that lowering blood glucose can effectively improve cardiovascular complications in patients with diabetes. Notably, several newly developed hypoglycemic drugs have been shown to exert a beneficial effect on the cardiovascular system beyond their ability to lower blood-glucose levels.

Glucagon-like peptide-1 (GLP-1) can stimulate endogenous insulin release. However, the half-life of GLP-1 is very short; once produced, it is degraded by dipeptidyl peptidase-4 (DDP-4) in short time [135]. New hypoglycemic agents, including DDP-4 inhibitors and GLP-1 receptor agonists, have been shown to have a beneficial effect on the cardiovascular system. The DPP-4 inhibitor sitagliptin is the first hypoglycemic agent to exhibit a comprehensive cardiovascular safety profile proven by a large randomized clinical trial [136]. The result of that study (TECOS) showed that sitagliptin therapy does not increase the incidence of cardiovascular endpoint events, which fully demonstrates its cardiovascular safety. According to the LEADER trial, the addition of the GLP-1 analog liraglutide to conventional treatment significantly reduced the incidence of cardiovascular end point events in patients with type 2 diabetes compared with the placebo group [137, 138].

Sodium-glucose cotransporter 2 (SGLT-2) inhibitors reduce the glucose concentration by selectively inhibiting SGLT2 in proximal renal tubules, thereby reducing glucose reabsorption and promoting glucose excretion. According to the EMPA-REG OUTCOME trial, an SGLT-2 inhibitor (empagliflozin) significantly reduced the risk of cardiovascular death in diabetes patients [139]. This result from EMPA-REG OUTCOME showed that patients with type 2 diabetes who were at high risk of cardiovascular events had a significantly reduced risk of cardiovascular death (lowered by 38%) when empagliflozin was added to the standard regimen [140]. The drug also reduced all-cause death risk (by 32%) and hospitalizations due to heart failure (by 35%) [141]. Animal studies revealed that empagliflozin treatment significantly reduces oxidative stress in cardiac tissues with no blood pressure reduction or improvement of cardiac autonomic dysfunction [142].

Currently, evidence of the effect of these new hypoglycemic agents on diabetic cardiovascular complications may be mediated through their ability beyond antidiabetic effects [143]. However, the direct mechanisms of these new hypoglycemic agents are not fully understood.

## 8. Conclusions

In summary, it is widely accepted that diabetes aggravates cardiovascular diseases and that patients with diabetic cardiovascular complications experience worse clinical outcomes. The abovementioned data suggest that AGE-RAGE axis inhibition or blockade of its interaction with oxidative stress is a novel therapeutic strategy for preventing cardiovascular complications in diabetes. The master role of AGEs and oxidative stress in cardiovascular complications of diabetes has been widely recognized, but many of these mechanisms are not yet clear and need further clarification since AGEs not only modulate oxidative stress but also in turn are affected by oxidative stress.

Fortunately, a series of drug candidates were reported to reduce oxidative stress and/or the AGE-RAGE axis. In addition, several new glucose-lowering drugs have also been found to exert a protective effect on the cardiovascular system beyond blood-glucose control in this population. A deeper study of these mechanisms and drugs will facilitate the designing of new drugs and provide new ideas for the prevention and treatment of diabetes and associated cardiovascular complications.

## Figures and Tables

**Table 1:** Therapeutic candidates against diabetic cardiovascular complications.

Category	Examples	Mechanisms of act
Traditional hypoglycemic agents	Metformin	Antioxidant properties and possible effects on the reduction of AGE

Antioxidants	Vitamin C and vitamin E	Antioxidant properties
	ACEIs and ARBs	Reduce angiotensin II-induced oxidative stress
	Statins	Reduce lipid peroxidation

AGE-RAGE inhibitors	ALT-711	AGE cross-link breaker
	Aminoguanidine	Inhibit AGE formation
	Soluble RAGE	Competitively combine with AGEs

New hypoglycemic agents	GLP-1 receptor agonists	Not fully understood
	DDP-4 inhibitors	Not fully understood
	SGLT-2 inhibitors	Not fully understood
